# A randomized control trial to assess optical coherence tomography parameters of the Xlimus drug-eluting stent: the XLIMIT trial

**DOI:** 10.3389/fcvm.2023.1199475

**Published:** 2023-09-06

**Authors:** Luca Testa, Mattia Squillace, Nicoletta Ventrella, Raul Moreno, Santiago Jiménez-Valero, Antoni Serra, Joan Antoni Gomez Hospital, Michele Bellamoli, Antonio Popolo Rubbio, Francesco Bedogni

**Affiliations:** ^1^ Department of Cardiology, IRCCS Policlinico S. Donato, Milan, Italy; ^2^ Department of Cardiology, Hospital La Paz, IdiPAZ, Madrid, Spain; ^3^ Department of Cardiology, Hospital de la Santa Creu i Sant Pau, Barcelona, Spain; ^4^ Department of Cardiology, Hospital Bellvitge, Barcelona, Spain; ^5^ Department of Cardiology, Fondazione Poliambulanza, Brescia, Italy

**Keywords:** third generation DES, neointimal volume, OCT, restenosis, endothelialization third generation DES

## Abstract

**Background:**

Third generation drug-eluting stents (DES) potentially offer better technical performance and reduced neointimal proliferation than previous generation DES. The XLIMIT non-inferiority trial evaluated the performance of the Xlimus (a novel sirolimus-eluting coronary stent system) in terms of endothelialization and tissue healing compared to the bioresorbable polymer Synergy DES.

**Methods:**

A total of 177 patients undergoing percutaneous coronary intervention (PCI) were randomized in a 2:1 ratio (2 Xlimus: 1 Synergy). The primary endpoints, defined as the in-stent neointimal volume weighted by the sum of the lengths of the implanted stent (ISNV) and the in-stent neointimal percent volume obstruction (%VO) were evaluated at 6–9 months by means of optical coherence tomography (OCT). Additional OCT parameters as well as clinical endpoints were also collected.

**Results:**

Most of the patients were males (77.4%), and the mean age was 64 years. One third of the population had stable angina/silent ischemia. A total of 300 stents (237 lesions) were analyzed: 198 (152 lesions) were in the Xlimus group, and 102 (85 lesions) in the Synergy group. The ISNV in the Xlimus group was 30.7 ± 24.5 mm^3^ while in the Synergy group it was 26.5 ± 26.7 mm^3^: the difference between the two means was 0.08 (−0, 04–0, 45), *p* = 0.018, thus meeting the non-inferiority hypothesis. The %VO was 16.3% ± 10.4% and 13.3% ± 10.8% in the Xlimus and Synergy groups, respectively: the difference between the two means was 3.0 (−0, 06–4, 2), (*p* = 0.01), thus meeting the non-inferiority hypothesis. No difference was found with respect to the secondary OCT endpoints as well as for clinical endpoints.

**Conclusions:**

The study results confirm that the biological interaction of the Xlimus and Synergy DES with the coronary artery is comparable, and that translates in very reassuring OCT parameters at follow-up: as such, the Xlimus is non-inferior to the Synergy.

**Clinical Trial Registration:**

ClinicalTrials.gov, identifier (NCT03745053).

## Introduction

Late stent thrombosis is an ominous complication of drug-eluting stent technologies (DES) (1–5) and is thought to be related to the lack of complete endothelialization and the chronic inflammatory stimulus caused by the permanent presence of a polymer on the stent surface (6).

With the introduction of second-generation DES, new polymers with lower impact on platelet activity, bioerodible polymers, and polymers with coating limited to the abluminal surface of the struts have been adopted in order to possibly reduce the incidence of late stent thrombosis (7–11).

The XLIMUS® sirolimus-eluting coronary stent system is an ultrathin device with an abluminal biodegradable polymer coating designed to ensure rapid reendothelialization.

This may lead to long term clinical advantages such as lower revascularization rate and eventually reduce clinically relevant events.

The Xlimit trial aimed to assess the safety and efficacy profile of the Xlimus drug-eluting stent and to compare it to the Synergy bioabsorbable polymer everolimus-eluting stent in patients undergoing percutaneous coronary intervention (PCI) over a 12-month follow-up (FU).

## Methods

### Study population

The XLIMIT study was designed as a multicenter randomized controlled trial (RCT): its rationale and design has been published previously (12).

Patients affected by stable or unstable angina, non-ST segment elevation myocardial infarction were considered for randomization if suitable for PCI.

Patients with ST-elevated myocardial infarction (MI), left main disease, chronic total occlusions (CTO), venous graft disease, in-stent restenosis (ISR), or recent (less than 3 months) coronary intervention on target vessels were excluded. Exclusion criteria included known hypersensitivity to heparin, aspirin, clopidogrel, ticlopidine, sirolimus, everolimus, or contrast media, pregnancy, history of bleeding or known coagulation disorders, left ventricle ejection fraction (LVEF) <30%, a life expectancy of <1 year, or impossibility of undergoing all follow-up examinations and procedures.

### Enrollment and data collection

A total of 177 patients were recruited from February 2019 to March 2021 at four investigational sites and randomized into the two groups in a 2:1 ratio (2 Xlimus: 1 Synergy).

At 6–9 months, all patients underwent an angiography and optical coherence tomography (OCT) evaluation as well as a clinical follow-up. The latter was repeated at 12 months.

This study was conducted in accordance with the ethical principles of the Declaration of Helsinki (2013). The protocol of this study was approved by the Ethics Committee of participating hospitals. Printed informed consent and detailed information about the study were offered to patients before randomization.

All the demographic and procedural data were collected in a web-based case report form (CRF) as well as the events recorded at FU.

Prior to the procedure, patients were pre-medicated according to local standard practices. As such, patients received aspirin and a loading dose of clopidogrel 600 mg, prasugrel 60 mg, or ticagrelor 180 mg, unless they were already taking an antiplatelet for at least 5 days prior to the procedure. Anticoagulants, antiplatelets, and coronary vasodilator therapies were administered following the current guidelines. After the index procedure, dual antiplatelet therapy (DAPT) was recommended for 6 to 12 months according to current medical guidelines.

Randomization was performed after the indication to PCI was given by means of an automatic response website. Patients were blind to the treatment, operators were aware. Procedures could be *ad hoc* or staged. The core lab was blinded.

### Devices description

The Synergy DES is made of a thin-strut (74–81 μm) platinum chromium (PtCr) metal alloy platform and a 4 μm bioabsorbable polylactic (PLGA) abluminal polymer which elutes the everolimus. Elution is complete by 90 days, and polymer absorption is essentially complete by 120 days [7–10] (13–16).

The stent platform of the XLIMUS is made of cobalt chromium L 605 and the stent is available in a 6-, 8-, or 10-cell structure design (closed cell architecture). The strut thickness is 73 μm and the 6-cell design is for the stenting of coronary arteries with a diameter of 2.25–2.50 mm; the 8-cell structure is used for the stenting of 2.75–3.50 mm diameter arteries; and the 10-cell is for larger artery diameter lesions (up to 5 mm). The XLIMUS has an innovative hydrophilic-coated shaft and an extra-low tip profile (crossing profile = 0.90 mm) to access the most tortuous lesions. Of note, within 30 days, about 70% of the sirolimus is distributed into the surrounding arterial tissue of the stent struts. Elution is complete by 90 days, and polymer absorption is essentially complete by 120 days.

### Coronary angiography assessment and optical coherence tomography analysis

OCT examinations of target vessels were performed after intracoronary administration of 200 μg of nitroglycerin. All OCT sequences were analyzed by an independent core laboratory using offline software (OPTISTM Imaging Software).

Conventional definitions derived from expert consensus OCT documents were applied (17–19). Analyses were performed by dividing the lesion length into quartiles and then by selecting three frames for the analysis: a distal frame between the first and the second stent quartile, one frame at mid-stent, and one proximal frame between the third and the fourth quartile. The assessment of neointimal thickness was calculated as the difference between the stent contour and the luminal contour. For each measurement, the values of the three frames were then averaged to derive the mean data. Volumetric measurements were obtained by applying the Simpson rule (20). Overlapping stents were considered as a single lesion.

### Study outcomes

**Figure 1:**
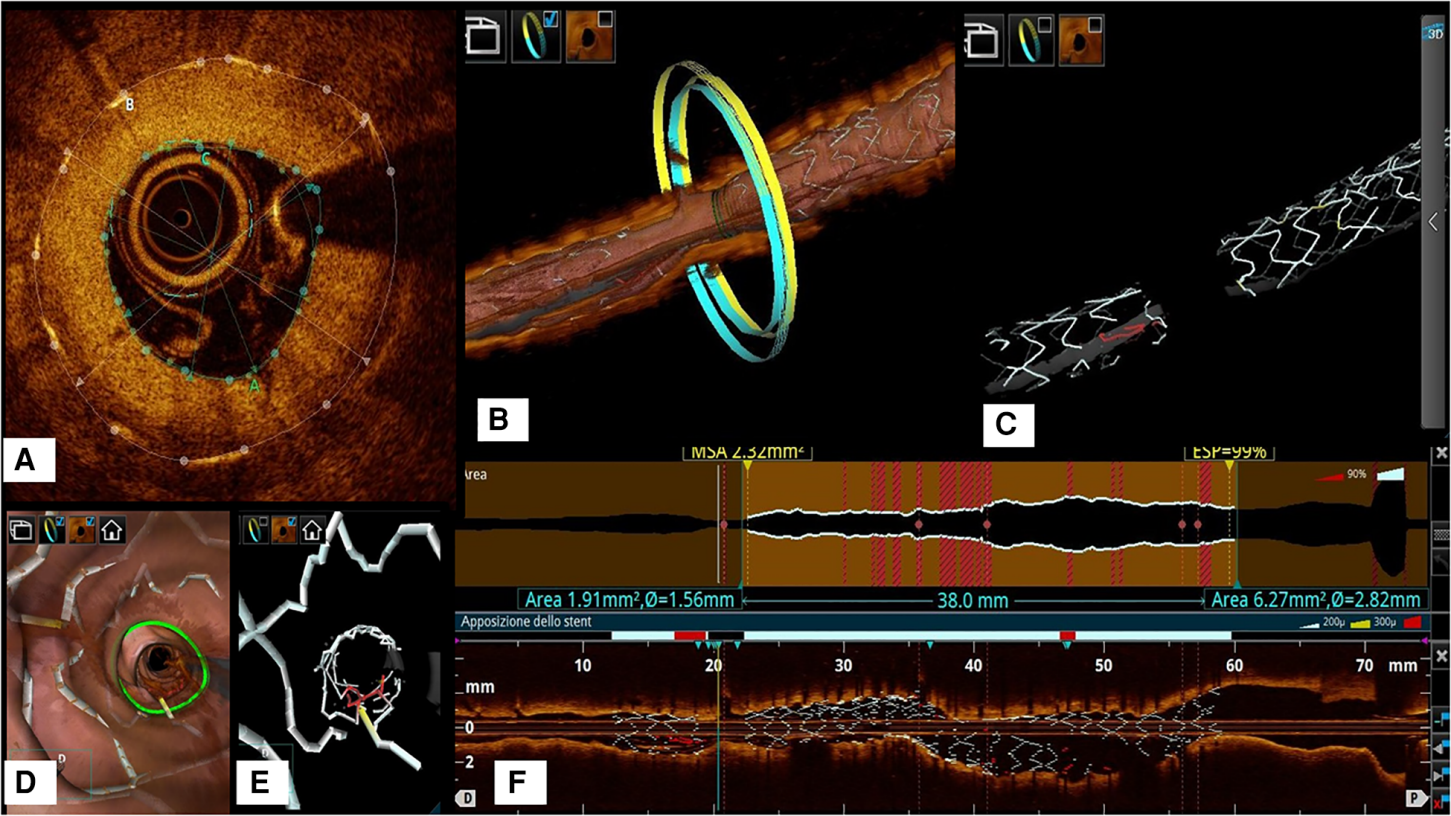
(**A**) Example of frame-level evaluation of neointimal thickness and lumen area which are key measures to derive both the co-primary OCT endpoints (%VO and ISNV). (**B**): 3D model showing the stent and the coronary artery at the frame considered in example A. (**C**): 3D model of the stent alone to show how the OCT represents the restenotic segment as a gap in the stent struts. (**D**): flythrough model to show the irregular lumen profile that correlates with the neointimal hyperplasia as a consequence of malapposed struts (in red in Panel **E**). (**F**): longitudinal reconstruction and lumen profile showing the narrowing of the artery at the restenotic segment.

The primary endpoints described below were evaluated by means of OCT at 6–9 months, according to international standards (17, 18):

(1) the in-stent neointimal percent volume obstruction [%VO, obtained by dividing the stent volume (mean stent area by stent length) by the in-stent neointimal volume and multiplied by 100], and
(2) Neointimal volume weighted by the sum of the lengths of the implanted stent (ISNV) (Figure 1).

Secondary outcomes included angiographic and clinical parameters, and in particular neointimal area calculated at the narrowest luminal area segment of target vessels, cardiovascular death, target vessel MI or target vessel failure (stent thrombosis, restenosis, or target vessel revascularization: TVF), ischemia-driven target lesion revascularization (TLR), stent thrombosis (ST), device success at 24 h, and procedural success at 24 h.

Definitions are as follows: device success at 24 h is defined as the deployment of the assigned stents without system failure or device-related complication (time frame: 24 h); procedural success at 24 h indicates lesion success without the occurrence of major adverse cardiovascular events (MACE) during the hospital stay (time frame: 24 h).

All clinical endpoints were adjudicated by an independent event adjudication committee (EAC) of interventional and non-interventional cardiologists who were not participants in the study.

### Sample size determination

Sample size was calculated and based on data obtained from historical cases in published databases including more than 1,500 lesions (21). We assumed a mean value of in-stent percent volume obstruction of 15% ± 7.5% in the everolimus DES group, and hypothesized a volume reduction of 4% with the Xlimus, leading to a mean of 11%. Thus, aiming for a two-tailed *α* of 0.05% and 80% power of Student's *t*-test, the required total sample was 129 patients (43 in the everolimus DES/Synergy group and 86 in the sirolimus DES group). This sample size had to be increased to 135 patients (45 in the everolimus DES/Synergy group and 90 in the sirolimus DES group) considering potential suboptimal image acquisition in 3% of cases. Finally, considering a 25%–30% cumulative rate of drop-outs, it was reasonable to enroll 60 patients in the everolimus DES/Synergy group and 120 in the novel Xlimus group to test the non-inferiority hypothesis at a significance level *α* of 0.05 one-tailed (equivalent to a 90% confidence interval) with a threshold for non-inferiority of 15%, and assuming that the two treatments are actually equivalent.

### Statistical methods

Descriptive statistics (arithmetic mean, median, minimum and maximum, and standard deviation) were calculated for quantitative variables. Absolute frequencies and percentages were obtained for qualitative variables. All statistical tests were performed as two-sided α = 0.05.

Student's *t*-test or Mann–Whitney *U* test were used to compare quantitative variables depending on whether they were normally distributed or not; *χ*2 or Fisher's exact tests were used to compare qualitative variables.

The primary endpoints were analyzed by means of the Student's *t*-test.

The probability of the non-occurrence of clinical secondary endpoints at 12 months (cardiac death, target-vessel MI and clinically indicated TLR, target-vessel MI, TLR, and ST) were estimated using the Kaplan–Meier method and compared between the two treatment groups by means of the log-rank test, followed by the use of the Cox proportional hazards model to assess the predictive models in consideration of the baseline characteristics statistically associated with the events and the following variables: treatment group, age, gender, number of vessels treated, stent length, number of stents, and insulin therapy requirement.

## Results

### Patients

Demographic and clinical features of the study population are summarized in Table 1. A total of 177 patients were enrolled: 117 in the Xlimus group and 60 in the Synergy group. The two groups were comparable in terms of both demographic and angiographic characteristics.

**Table 1:** Demographic and clinical features.

	Xlimus (117 pts)	Synergy (60 pts)
Age (mean)	62 (10)	63 (10)
Gender % (M/F)	82/18	84/16
BMI mean (SD)	27 (3.9)	26 (3.6)
Hypertension	75%	80%
Diabetes	30%	26%
Hypercolestherolemia	54%	52%
Smoking habit	30%	33%
Family history of CAD	33%	40%
Prior MI	24%	18%
Prior PCI	30%	28%
COPD	4%	5%
Atrial fibrillation	3%	5%
CKD >2grade (GFR <60 ml/min)	2%	2%
TIA/stroke	5%	3%
LVEF <40%	8%	7%
Stable angina/inducible ischemia	31%	28%
UA/NSTEMI	69%	72%
Multivessel disease	45%	40%
Aspirin	98	97
DAPT (at the time of PCI)	90	90
Beta blockers	40	38
Calcium antagonist	18	20
ACE/ARBs	66	70
Lipid lowering therapy	68	66
Raised CK-MB, TnI (% of patients)	40	38

BMI, body mass index; CAD, coronary artery disease; MI, myocardial infarction; PCI, percutaneous coronary intervention; COPD, chronic obstructive pulmonary disease; CKD, chronic kidney disease; TIA, transient ischemic attack; LVEF, left ventricle ejection fraction; UA, unstable angina; NSTEMI, non-ST elevation myocardial infarction; DAPT, dual antiplatelet therapy; ACE, angiotensin converting enzyme; ARB, angiotensin receptor blocker.

Almost one-third of the patients had diabetes, while almost 20% of the patients had a previous myocardial infarction.

At inclusion, two-thirds of the population suffered from an acute coronary syndromes (ACS). A severely depressed LVEF concerned a minority of the population.

Aspirin was taken by 65.3% of patients and antiplatelets in 24% of cases. Statins were taken by a large proportion of patients (61.9%) and beta-blockers by 44.9%. Dual antiplatelet therapy was prescribed in 98.3% of patients.

### Procedural data

Angiographic and procedural data are listed in Table 2. A radial approach was adopted in 95% of cases. Mean diameter stenosis was 82.50% ( ± 9.6%) and 82.22% ( ± 11.23%), mean lesion length was 21.80 ( ± 11.46) mm and 22.98 (± 14.03) mm, and mean RVD was 2.93 (±0.52) mm and 2.95 (± 0.45) mm in the Xlimus and Synergy groups, respectively.

**Table 2:** Angiographic and procedural data.

	Xlimus (117 pts)	Synergy (60 pts)	*p*
Radial access	95%	94%	0.2
Multivessel disease	45%	40%	0.2
Culprit lesion (LAD/Diag)	50%	48%	0.2
Culprit lesion (LCx/OM)	26%	28%	0.3
Culprit lesion (RCA)	24%	24%	1
Culprit lesion length, mm (mean/SD)	21 (11)	22 (14)	0.4
Treated lesion (LAD/Diag)	55%	55%	–
Treated lesion (LCx/OM)	24%	28%	0.2
Treated lesion (RCA)	21%	17%	0.3
Diameter stenosis % (mean, SD)	82 (9)	82 (11)	0.5
Reference vessel diameter, mm (mean/SD)	2.9 (0.5)	2.9 (0.4)	0.2
Total number of implanted stents	198	102	0.4
Number of implanted stents per patient (min-max)	1.7 (1–3)	1.6 (1–3)	0.3
Number of implanted stents per lesion	1.3	1.2	0.5
Stent length (mean/SD)	22.58 (11.94)	23.39 (14.11)	0.7
Stent diameter (median, IQR)	3 (2.5–3.5)	3 (2.5–4)	0.1
Pre-dilatation (%)	95 (81%)	48 (80%)	0.3
Max balloon diameter (median, IQR)	2.5 (2–3)	2.5 (2–3)	0.7
Max balloon inflation pressure (median, IQR)	12 (8–20)	12 (8–22)	0.2
Post-dilatation (%)	85 (73%)	48 (80%)	0.3
Max balloon diameter (median, IQR)	3.5 (3–3.75)	3.5 (3–3.5)	0.2
Max balloon inflation pressure (median, IQR)	20 (10–26)	20 (08–26)	0.2
Procedural success at 24 h	192 (97%)	100 (98%)	0.1
Device success at 24 h	196 (99%)	102 (100%)	0.2
Residual syntax score (mean; median)	3.91 (4.96)	2.79 (3.43)	1
Residual stenosis >20%	0%	0%	1

IQR, interquartile range.

A total of 237 lesions were treated, 152 in the Xlimus group and 85 in the Synergy group; 198 and 102 stents were implanted in the two groups, respectively.

Device success at 24 h was achieved in 98.7% and 100% of cases in the Xlimus and Synergy groups, respectively.

Procedural success was obtained in 97% and 98% of the cases, respectively.

There were no device malfunctions.

At discharge, 100% of patients were asymptomatic. Most patients (96.6%) were prescribed statins and betablockers (74.1%).

### Primary endpoint

The primary analysis concerned a total of 300 DES (198 Xlimus and 102 Synergy) in 177 patients (117 in the Xlimus group vs. 60 in the Synergy group), evaluated by means of angiography at 6 to 9 months after the index procedure.

No statistically significant difference was observed in either of the two OCT-derived primary endpoints. In particular, the %VO was 16.3% ( ± 10.49%) and 13.3% ( ± 12.88%) for the Xlimus DES and Synergy DES, respectively: the difference between the two means was 3.0 (−0,06–4,2), (*p*** **=** **0.01), thus meeting the non-inferiority hypothesis.

**Figure 2:**
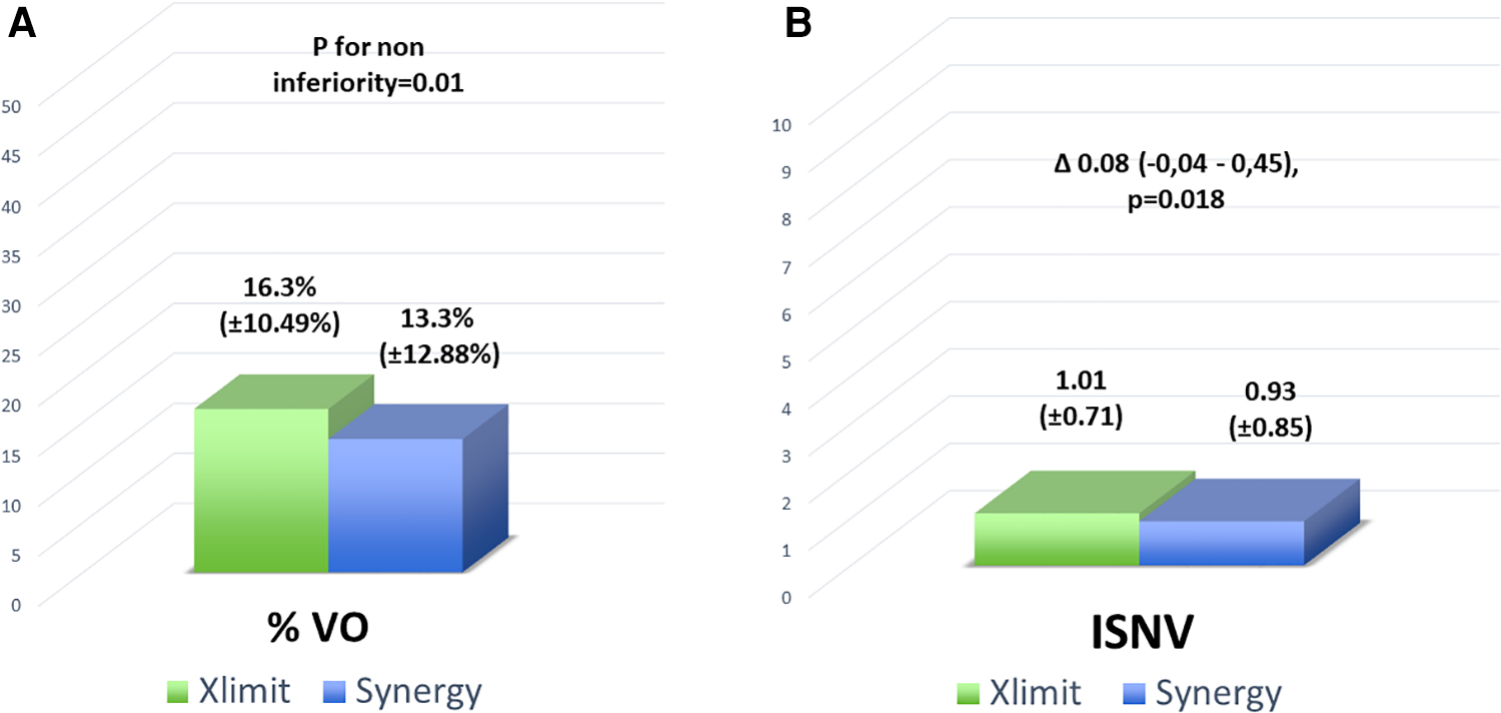
Co-primary endpoints: (**A**) the in-stent neointimal percent volume obstruction [%VO, obtained by dividing the stent volume (mean stent area by stent length) by the in-stent neointimal volume and multiplied by 100], and (**B**) neointimal volume weighted by the sum of the lengths of the implanted stent (ISNV).

Moreover, the ISNV was 1.01 ( ± 0.71) and 0.93 ( ± 0.85) mm^2^ for the Xlimus DES and Synergy DES, respectively: the difference between the two means was 0.08 (−0,04–0,45), *p* = 0.018, thus meeting the non-inferiority hypothesis (Figure 2).

### Secondary endpoints

OCT/Angio FU was done at 240 ± 18 days and 233 ± 20 days, *p* = 0.3, in the Xlimus group and Synergy group, respectively.

The secondary OCT parameters are listed in Table 3.

**Table 3:** Secondary OCT parameters.

	Xlimus (*N* = 117 pts, 198 stents)	Synergy (*N* = 60 pts, 102 stents)	*p*
Neointimal area calculated at the site of minimal lumen area (mm^2^)	1.42 (1.10)	1.26 (1.35)	0.4
Stent area (mm^2^)	7.24 (2.52)	7.58 (2.64)	0.4
Lumen area (mm^2^)	6.22 (2.58)	6.68 (2.45)	0.3
MLA (mm^2^)	4.25 (1.94)	4.69 (1.93)	0.2
Minimal stent area (same cross-section of MLA)	5.79 (2.26)	6.01 (2.11)	0.5
Covered struts (%)	99	98	0.2
Apposed struts (%)	95	96	0.3
Neointimal hyperplasia thickness per struts, µ (mean, SD)	55 (22)	59 (18)	0.4
Incomplete stent apposition distance, µ (mean, SD)	188 (100)	194 (98)	0.2

Clinical endpoints occurred in a low number of cases and are listed in Table 4.

**Table 4:** Clinical endpoints.

	Xlimus (*N* = 117 pts, 198 stents)	Synergy (*N* = 60 pts, 102 stents)	*p*
Cardiac death, target vessel MI and TLR at 12 months	11 (9%)	4 (6.7%)	0.09
Cardiac death at 12 months	1 (1%)^a^	0 (0.0%)	0.4
Target vessel MI at 12 months	2 (2%)	1 (1.7%)	0.6
ID-TLR at 12 months	8 (6.8%)	3 (5.0%)	0.09
Stent thrombosis	0	0	–

^a^ Fatality occurred 11 months post the index procedure for acute MI of non-culprit vessel.

The variables statistically associated with the composite MACE occurrences are the procedural success at 24 h (*p* = 0.0058: HR = 0.127; 95% CI: 0.029–0.550), and the device success at 24 h (*p* = 0.0009; HR: 0.024; 95% CI: 0.003–0.219).

No statistically significant association with MACE was found for sex (*p* = 0.2637), age (*p* = 0.2738), COPD (*p* = 0.9894), hypertension (*p* = 0.5452), hypercholesterolemia (*p* = 0.2774), familiarity (*p* = 0.2363), diabetes (*p* = 0.1046), and smoking at a borderline value (*p* = 0.0772).

Similarly, the number of lesions and severe calcification were not associated with MACE (respectively, *p* = 0.18; HR = 0.437 for >1 vs.1, 95% CI: 0.127–1.501; and *p* = 0.25; HR = 2.362 for severe vs. no, 95% CI: 0.545–10.227).

## Discussion

The XLIMIT randomized controlled trials showed that the Xlimus sirolimus-eluting stent features a similar performance in terms of endothelialization process, assessed by means of OCT, to the Synergy everolimus-eluting stent.

In particular, both the neointimal volume weighted by the length of the implanted stents as well as the in-stent neointimal percent volume obstruction met the non-inferiority hypothesis.

Synergy EES is one of the new generation of thin-strut stents with abluminal bioabsorbable polymer and has shown good clinical and procedural results in several studies (13–15).

Some of the feature of the Xlimus are plausibly the reason for this performance: the high rate of device success proves the high deliverability of the Xlimus drug-eluting stent. The latter is conceivably related to the innovative hydrophilic-coated shaft and the extra-low tip profile that allows the stent to cross the most tortuous and calcified lesions.

The highly biocompatible PLLA (polylactid acid) matrix degrades smoothly and provides an optimal release kinetic profile. Within 30 days, about 70% of the anti-proliferative drug is distributed into the surrounding arterial tissue by the abluminal stent struts, ensuring a highly effective inhibition of smooth muscle cell migration and proliferation. The OCT follow-up showed that all the stents were fully endothelialized without any signal of excessive neointimal proliferation.

Indeed, the Xlimus DES performance in terms of %VO (16.3% ± 10.4%) is similar, if not better, to the reported percentage of the Mistent and Xience (MiStent 14.54% ± 3.7% and Xience 19.11% ± 6.70%) (16, 21). Of note, the consistency of the %VO evaluated in this study with the reported data of the DESSOLVE III study suggest the quality and reproducibility of the OCT analysis, which is often a major concern of all OCT studies (17–19).

Although it was in a relatively small population, long lesions and severe calcification were not associated with MACE, thus suggesting that the endothelialization process was effective also in more complex lesions. This finding is hypothesis-generating only and needs to be evaluated further.

### Safety

No stent thrombosis was detected in all the study population. It is known that impaired arterial healing after stent implantation is associated with a higher incidence of stent thrombosis (20, 22). The pathogenic mechanism seemed to be related to the polymer-related inflammatory reaction and endothelial cell dysfunction, which may cause predisposition to more thrombus formation on uncovered struts and, later on, to accelerate the neo-atherosclerosis (6, 23, 24).

The complete, predictable, and fast endothelialization might be taken as a surrogate to hypothesize the safety of a short DAPT regimen. This would certainly require a dedicated study.

In both groups, we found a low incidence of MACE (9% Xlimus vs. 6.7% Synergy) and ischemia-driven TLR (6.8% Xlimus vs. 5% Synergy).

TLR in both groups was comparable with the available literature on third generation DES (25, 26): this is a positive signal considering that a third of the population was diabetic and about 70% had an acute coronary syndrome at presentation.

### Study limitation

The study is underpowered to draw any meaningful clinical considerations. As such, whether the technical features of the Xlimus DES may translate into a clinical advantage against previous generation DES has to be evaluated in an adequately sized trial.

This trial was performed, especially the follow-up, during the COVID-19 pandemic, thus the recruitment and data collection was much slower than expected: however, the very high quality of the OCT recordings and the commitment of the enrolling centers made possible the evaluation of a higher-than-expected number of cases, considering that the sample size assumptions included a significant rate of dropouts that we ultimately did not observe.

## Conclusions

The Xlimus sirolimus-eluting stent showed to be comparable to the Synergy everolimus-eluting stent in terms of reendothelization process and arterial healing, as well as in terms of safety. A larger, clinically oriented trial would strengthen the good existing data.

## Data Availability

The datasets presented in this article are not readily available. Any request will be evaluated according to its scope. Requests to access the datasets should be directed to: luctes@gmail.com.
